# Supplementary material for: Cascade Screening and Treatment Initiation in Young Adults with Heterozygous Familial Hypercholesterolemia
Source: J Clin Med. 2021 Jul 13;10(14):3090. doi: 10.3390/jcm10143090 (PMC8306062; doi:10.3390/jcm10143090)
Supplement: Supplementary file 1 [file jcm-10-03090-s001.zip › jcm-1282903-supplementary.pdf]

Table S1: Survey Questions

1. Are you a US-based healthcare provider who currently treats patients with elevated LDL cholesterol?

   a. Yes

   b. No – **Out of survey if No is selected**

2. Are you licensed to prescribe medication in the United States?

   a. Yes

   b. No – **Out of survey if No is selected**

3. Please select your medical discipline:

   a. Physician

   b. Nurse Practitioner - **Out of survey if selected**

   c. Physician Assistant - **Out of survey if selected**

   d. Other (specify) _______________ - **Out of survey if selected**

4. Which best describes your medical area of practice? Select One

   a. Family Practice

   b. General Practitioner

   c. Internal Medicine

   d. Cardiology

   e. Other (specify) _______________ **- Out of survey if selected**

5. Please select the state where you primarily practice:

   a. Drop down list of 50 states.

6. Are you a lipid specialist as defined by having an expertise in complex lipid management and routinely have patients referred to you for their lipid management?

   a. Yes – **Out of survey if Yes is selected**

   b. No

7. Have you seen any patient who before being prescribed lipid lowering therapy had an LDL cholesterol ≥ 190 mg/dL?

   a. Yes

b. No – **Out of survey if No is selected**

8. Which best describes your practice location? Select One

a. Rural

b. Suburban

c. Urban

9. Which best describes your practice setting? Select One

a. Academic Institution

b. VA/Government Health System

c. Health System

d. Private Practice

e. Other

10. What would you recommend as initial treatment for a 30-year old normal weight male with a family history of premature coronary heart disease who despite life-style changes has an LDL cholesterol of 230 mg/dL (triglycerides are normal)?

a. Low to moderate intensity statin

b. High intensity statin

c. Improved diet and exercise and repeat lipid panel in 6 months

d. Ezetimibe

11. What is your most likely diagnosis for the patient described above?

a. Homozygous Familial Hypercholesterolemia

b. Heterozygous Familial Hypercholesterolemia

c. Mixed Hyperlipidemia

d. Familial Chylomicronemia

e. High cholesterol due to poor lifestyle habits

12a. Have you ever diagnosed a patient with an LDL cholesterol $\geq$ 190 mg/dL as having Heterozygous Familial Hypercholesterolemia (HeFH)?

a. Yes

b. No

12b. **(IF ANSWERED YES TO # 12a**) Which method(s) have you used to come to that diagnosis? Rank in order of method, with 1 being most preferred and 4 being least preferred.

a. Genetic testing

b. Family history of premature cardiovascular disease or high cholesterol

c. Level of LDL cholesterol

d. Dutch Lipid Clinical Network Score or Simon Broome Diagnostic Criteria

12c. **(IF ANSWERED YES TO # 12a)** What is your initial step in managing a patient that you have diagnosed with HeFH?

a. Lifestyle changes alone and repeat lipid profile in 6 months

b. Prescribe medication

c. Refer the patient to a lipid specialist

12d: **(IF ANSWERED NO TO # 12a)** What is the reason that you have never diagnosed a patient with an LDL cholesterol ≥ 190 mg/dL as having HeFH?

a. I am unfamiliar with the criteria for diagnosing HeFH
b. Unless the patient has cutaneous xanthomas, I am not confident in making the diagnosis
c. Unless the patient has a positive genetic test, I am not confident in making the diagnosis
d. Unless a family history of premature cardiovascular disease is present, I am not confident in making the diagnosis

13a. On a scale of 1 to 5, with 1 being "Not At All Familiar" and 5 being "Very Familiar", how would you rate how familiar you are with the Dutch Lipid Clinical Network Score to diagnose HeFH?

13b. On a scale of 1 to 5, with 1 being "Not At All Familiar" and 5 being "Very Familiar", how would you rate how familiar you are with the Simon Broome Diagnostic Criteria to diagnose HeFH?

13c. **(IF ANSWERED 2 -5 on scale for question 13a)** On a scale of 1 to 5, with 1 being "Never" and 5 being "Always", how often do you use the Dutch Lipid Clinical Network Score to diagnose HeFH?

13d. **(IF ANSWERED 2 -5 on scale for question 13b**) On a scale of 1 to 5, with 1 being "Never" and 5 being "Always", how often do you use the Simon Broome Diagnostic Criteria to diagnose HeFH?

14. Does your practice have access to a lipid specialist?

a. Yes

b. No

15. Do you use a risk calculator to assess cardiovascular risk in a patient with HeFH?

a. Yes

b. No

16. In a patient with HeFH what risk factors would you use to determine cardiovascular disease risk? Check all that apply. (Programming note: randomize options on screen so no order bias; anchor the "None of the above" option at bottom)

a. LDL cholesterol

b. Non-HDL cholesterol

c. Coronary Artery Calcium (CAC) Scoring

d. Lipoprotein(a)

e. hsCRP

f. Family History

g. Positive genetic testing for HeFH

h. None of the above since they do not require further risk stratification (if they choose (h) then the rest should be blacked out)

17. In an adult patient with HeFH and an untreated LDL cholesterol ≥ 190 mg/dL, which of the following therapies would you recommend to the patient? Rank your top 5 choices in order of importance, where "1" is the most important, "2" is the second most important, etc.". (Programming note: randomize options on screen so no order bias)

a. High dose statin

b. Low to moderate dose statin

c. Ezetimibe

d. Fibrate

e. Bile acid sequestrant

f. Niacin

g. Plant sterols

h. Diet and exercise

i. Dietary supplements

j. Prescription fish oil

k. PCSK9 inhibitor

l. LDL apheresis

18. In an adult patient with HeFH on a maximally tolerated statin who needs additional LDL cholesterol lowering what would you use to lower the cholesterol concentration? Rank your top 5 choices in order of importance, where “1” is the most important, “2” is the second most important, etc.”. (Programming note: randomize options on screen so no order bias)

a. Ezetimibe

b. Fibrate

c. Bile acid sequestrant

d. Niacin

e. Plant sterols

f. Diet and exercise

g. Dietary supplements

h. Prescription fish oil

i. PCSK9 inhibitor

j. LDL apheresis

19. What treatment goal would you use for an adult HeFH patient who is free of clinical atherosclerotic cardiovascular disease (ASCVD)?

a. LDL cholesterol decrease of at least 50% from baseline

b. LDL cholesterol < 70 mg/dL

c. LDL cholesterol < 100 mg/dL

d. LDL cholesterol < 130 mg/dL

20. At what patient age do you typically begin monitoring LDL cholesterol levels in adult patients with a family history of high cholesterol or premature coronary heart disease?

a. 18 to 29 years old

b. 30 to 39 years old

c. > 40 years old

21. Do you treat patients under the age of 18?

a. Yes

b. No

22. On a scale of 1 to 5, with 1 being "Never" and 5 being "Always", how often do you recommend measuring cholesterol in the first-degree family members of a patient with HeFH ?

23a. At what age would you recommend measuring cholesterol in children of a patient of yours with HeFH?

a. 2 to 8 years old

b. 9 to 11 years old

c. 12 to 18 years old

d. I would not measure the cholesterol level until they were adults

23b. **(ANSWERED d FOR QUESTION 23a)** What are your reasons for not recommending measurement of cholesterol in the children of a patient of yours with HeFH? Choose all that apply.

a. I am not familiar with the guidelines for managing cholesterol in children

b. There is insufficient evidence that shows screening children for high cholesterol prevents ASCVD in adulthood

c. The children are not patients of mine

d. There are no safe treatment options to treat high cholesterol during childhood.

24. What age would you start a male patient with HeFH on statin therapy?

a. Under 18 years of age **(PROGRAMMING NOTE: NO HIDING BASED ON PREVIOUS QUESTIONS)**

b. 18-29 years of age

c. 30-39 years of age

d. 40 years of age or older

25. What age would you start a female patient with HeFH on statin therapy?

a. Under 18 years of age **(PROGRAMMING NOTE: NO HIDING BASED ON PREVIOUS QUESTIONS)**

b. 18-29 years of age

c. 30-39 years of age

d. 40 years of age or older

26. Do you currently have an HeFH patient on a PCSK9 inhibitor (alirocumab or evolocumab)?

a. Yes

b. No

27. On a scale of 1 to 5, with 1 being "Not At All Likely" and 5 being "Extremely Likely", how likely are you to prescribe a PCSK9 inhibitor in an adult patient with HeFH WITH clinical ASCVD on a maximally tolerated statin who needs additional LDL cholesterol lowering therapy?

28. On a scale of 1 to 5, with 1 being "Not At All Likely" and 5 being "Extremely Likely", how likely are you to prescribe a PCSK9 inhibitor in an adult patient with HeFH WITH NO clinical ASCVD on a maximally tolerated statin who needs additional LDL cholesterol lowering therapy?

29a. Have you ever personally prescribed a PCSK9 inhibitor to an adult patient with HeFH?

a. Yes

b. No **(IF ANSWERED NO THEN PROCEED TO QUESTION 29b AND SKIP QUESTION 30a and 30b)**

29b. What are the reasons you have never prescribed a PCSK9 inhibitor? Rank your choices in order of importance, where "1" is the most important, "2" is the second most important, etc.". (Programming note: randomize options on screen so no order bias)

a. There is no outcome evidence to support the use of a PCSK9 inhibitor in HeFH

b. The process is too difficult and time consuming

c. The medication is too expensive

d. I lack experience in prescribing a PCSK9 inhibitor

30a. On a scale of 1 to 5, with 1 being "Not At All Difficult" and 5 being "Extremely Difficult", have you encountered difficulty in the approval process when prescribing a PCSK9 inhibitor for a patient with HeFH?

30b. What barriers have you encountered when prescribing a PCSK9 inhibitor for an adult patient with HeFH? Rank your choices in order of importance, where "1" is the most important, "2" is the second most important, etc.". (Programming note: randomize options on screen so no order bias)

a. The pre-authorization process is very complicated

b. The pre-authorization process is time consuming

c. It is difficult to document a clinical diagnosis of HeFH

d. My patient does not want to take an injectable medication

e. The medicine is too expensive

f. I get frequent denials from the insurance company
